# Lipase Catalysed Kinetic Resolution of Racemic 1,2-Diols Containing a Chiral Quaternary Center

**DOI:** 10.3390/molecules23071585

**Published:** 2018-06-29

**Authors:** Gonzalo de Gonzalo

**Affiliations:** Departamento de Química Orgánica, Universidad de Sevilla, c/Profesor García González 1, 41012 Sevilla, Spain; gdegonzalo@us.es; Tel.: +34-954559997

**Keywords:** biocatalysis, lipases, kinetic resolution, 1,2-diols

## Abstract

Optically active 1,2-diols are valuable buildings blocks in organic synthesis. In the present paper, a set of racemic 1,2-diols with an ester functional group are prepared, starting from α-ketoesters in a three-step procedure with moderate yields. The racemic 1,2-diols, containing a chiral quaternary center in their structure, are subjected to selective acylation in order to perform their kinetic resolution catalysed by a set of commercially available lipases. Under optimized reaction conditions, good conversions and enantioselectivities are achieved by using the lipase PSL-C from *Pseudomonas cepacia* in *tert*-butyl methyl ether. This biocatalyst could be reused up to five times without losing its properties.

## 1. Introduction

Optically active 1,2-diols are valuable compounds as they can be transformed into several interesting molecules [1,2,3]. Due to their importance as building blocks in organic synthesis, a number of synthetic methodologies have been developed for their preparation [4,5]. One of the most widely applied methods is Sharpless asymmetric dihydroxylation, which involves the oxidation of alkenes to form 1,2-diols in presence of chiral catalysts [6,7]. This methodology suffers some limitations including the relatively low activity and selectivity of the aromatic compounds and the substrate scope. For these reasons, different alternatives have been investigated. The use of biologically active systems as catalysts in organic reactions, including whole cells, cells free extracts, or purified enzymes, has emerged as a powerful tool for the preparation of high added value compounds under mild and environmentally friendly conditions [8,9,10,11].

The enzymes that have been used to catalyse the formation of chiral 1,2-diols belong to the oxidoreductases (alcohol dehydrogenases and dioxygenases) [12,13], and the hydrolases, including epoxide hydrolases [14,15] and lipases. This last group of enzymes (EC 3.1.1.3) have demonstrated their synthetic applicability, being the most used type of enzymes in industrial chemistry [16,17,18]. Lipases are widely available, have broad substrate acceptance and are able to catalyse reactions not only in aqueous mediums, but also in organic solvents, which expands their synthetic repertoire. In addition, lipases usually display a high degree of chemo-, regio- and/or enantioselectivity in the processes that they catalyse.

Lipases have been used to prepare optically active 1,2-diols catalysing the kinetic resolution of racemic mixtures in acylation reactions, thus leading to chiral diols and esters that can be converted back to the starting diols via hydrolysis [19,20,21,22]. In 2000, the kinetic resolution of racemic ethyl 2-benzyl-2,3-dihydroxypropanoate was described, a 1,2-diol containing an ester moiety precursor in the synthesis of (*R*)-etomoxir, which is a powerful hypoglucemic reagent [23]. After testing different biocatalysts, the lipase PS from Amano was found to catalyse the formation of (*R*)-1,2-diol and (*S*)-acetate with high selectivity and activity. Given this result, we decided to synthesise a set of functionalised 1,2-diols with an ester group and perform their kinetic resolution in the presence of different lipases, with the aim of obtaining these valuable optically active compounds.

## 2. Results and Discussion

### 2.1. Preparation of the Racemic 1,2-Diols (±)-***1–6d***

The racemic 1,2-diols were prepared in a three-step procedure starting from the corresponding α-ketoesters **1**–**6a**, as indicated in Table 1. These compounds were treated with *N*-*tert*-butyl formaldehyde hydrazone in toluene at room temperature to yield the racemic azocompounds (±)-**1**–**6b** with high yields (75–92%) after 24 h. For almost all the ketoesters, the reaction occurred in the absence of any catalyst, but for ethyl benzoylformate (**1a**), the addition was accelerated in the presence of the Schreiner’s thiourea (**I**) at 10 mol%. The resulting azocompounds are valuable synthons that can be transformed in different compounds [24,25,26]. Their hydrolysis in a biphasic system Et_2_O/HCl (aq) yielded the corresponding hydroxyaldehydes (±)-**1**–**6c** after four hours via a tautomerization and hydrolysis process. Debt to their instability were directly reduced without purification to the racemic 1,2-diols (±)-**1**–**6d** by treatment with a mild reductant as tetrabutylammonium borohydride (NBu_4_BH_4_) in CH_2_Cl_2_ at room temperature over two hours. Depending on the substrate structure, the 1,2-diols were obtained with yields from 41 to 57%. Attempts to improve these yields by modifying certain reaction parameters as the hydrolysis conditions or the reducing agent were unsuccessful.

### 2.2. Kinetic Resolution of Racemic Diols (±)-***1***–***6d***

Once the racemic 1,2-diols were synthesized, their kinetic resolution was performed. Our initial studies were performed using racemic ethyl 2,3-dihydroxy-2-phenylpropanoate (±)-**1d** as the model substrate. The selective acetylation of this 1,2-diol (0.15 M) in toluene at 30 °C was performed in presence of 3.0 equivalents of vinyl acetate to ensure an irreversible acylation process. The effects of different biocatalysts were analysed by screening several commercially available lipases, as indicated in Table 2. For all the biocatalysts tested, the (*S*)-enantiomer of the 1,2-diol was acetylated, yielding (*S*)-**1e**, whereas the (*R*)-1,2-diol remained unaltered. The use of immobilized *Candida antarctica* lipase B (CalB) resulted in a reaction without selectivity (entry 1), achieving a 23% conversion after four hours. When the reaction was catalysed by its isozyme A (entry 2), a more selective process was observed (enantioselectivity, *E* = 12), demonstrating acylation slower than with CalB. As shown in entry 3, *Pseudomonas cepacia* lipase (PSL-C) seems to be the most suitable biocatalyst for this reaction, as a moderate *E* value was obtained in a process with a 32% conversion after 20 h, achieving (*S*)-**1d** with 86% *ee*. The use of lipases from *Pseudomonas fluorescens* (PSF), *Burkholderia* sp. (BSL), *Rhizopus oryzae*, and *Aspergillus oryzae* led to very low enantioselectivities (*E* < 10, entries 4–7), with conversions varying from 41% after 20 h with PSF, to 16% with the same duration using the lipase from *Aspergillus oryzae*. The kinetic resolution catalysed by porcine pancreatic lipase (PPL) in toluene afforded a selectivity value of 13, and a conversion close to 50% after 24 h, as shown in entry 8. The opposite lipase from *Mucor miehei* was not a suitable biocatalyst for this reaction, as only a 13% of (*S*)-**1e** was obtained after 24 h in a very low selective resolution (entry 9).

After selecting PSL-C as the best biocatalyst for the acetylation of racemic **1d**, we analysed other parameters that can affect the activity and selectivity of the biocatalyst. Thus, different organic solvents were tested in the acylation reaction. As shown in entry 10, *tert*-butyl methyl ether (TBME) was the best solvent for this process, as a good selectivity value could be achieved (*E* = 41) in a reaction much faster than in toluene (34% conversion after eight hours with 91% *ee* for **1e**). This solvent was also tested in the CalA-catalysed acylation, promoting an increase in both the enzyme activity and selectivity (entry 11). However, the *E* value remained low. The reaction catalysed by PPL in TBME afforded the chiral acetate (S)-**1e** with 77% *ee* in a process with a 38% conversion after 12 h (entry 12). Other solvents analysed in the acetylation catalysed by PSL-C, such as 1,4-dioxane or THF, led to slower resolutions and especially for the latter, whereas the enantioselectivities were around 15. The use of diisopropyl ether (DIPE) afforded (*R*)-**1d** with 67% *ee* and (*S*)-**1e** with 83% *ee* in a process with a 45% conversion after 12 h (entry 15).

The effect of temperature was also analysed in this kinetic resolution, performing the PSL-C-catalysed reaction of (±)-**1d** at 15 °C, as shown in entry 16. Lowering the temperature had no effect on the enzyme selectivity, whereas, as expected, the enzyme activity dropped (*c* = 25% after 12 h). The use of a less reactive acyl donor, such as isopropenyl acetate (entry 17), led to a similar selectivity. Chiral acetate (*S*)-**1e** was obtained with a 36% conversion and 91% *ee* after 16 h. When ethyl acetate was used as acyl donor (entry 17), a slower kinetic resolution was achieved, as 48 h were required to obtain a 34% yield of (*S*)-**1e** with 91% *ee* (entry 18).

**Figure 1:**
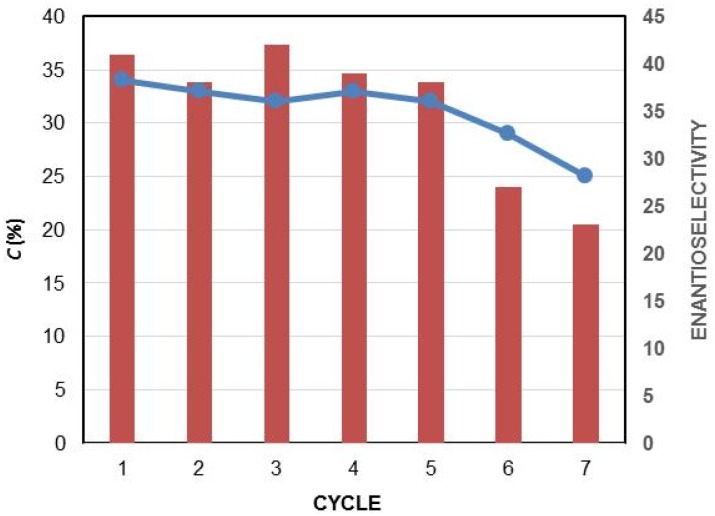
Effect of the PSL-C recycling on the conversion (blue line) and on the enantioselectivity (red bar) in the catalysed kinetic resolution of (±)-**1d** in *tert*-butyl methyl ether (TBME) at 30 °C using vinyl acetate as he acyl donor.

The recycling of the PSL-C was studied in the selective acetylation of (±)-**1e** with vinyl acetate in TBME at 30 °C. After 20 h, the biocatalyst was filtered, washed with TBME, and used again in a new reaction cycle. As shown in Figure 1, this biocatalyst could perform the selective acylation of the racemic diol for five cycles while maintaining its activity and selectivity. For the sixth reaction, a significant drop in the enantioselectivity of the process was observed (*E* = *27*). For the seventh reaction, this drop was accompanied by an appreciable loss in enzymatic conversion.

After the optimized conditions were set up for the kinetic resolution of racemic ethyl 2,3-dihydroxy-2-phenylpropanoate, using PSL-C and CalA in TBME at 30 °C and vinyl acetate as acyl donor, the scope of the reaction using different aromatic 1,2-diols was studied (Table 3). For all the substrates, (*S*)-acetates **2**–**6e** were the obtained products. The use of PSL-C led to higher enatioselectivities in all the aromatic and the heteroaromatic substrates (**2**–**5a**), whereas CalA showed higher activity. Thus, the enzymatic acylation of racemic methyl 2,3-dihydroxy-2-phenylpropanoate (**2d**) catalysed by PSL-C occurred with the same selectivity as for the ethyl analogue **1d** (*E* = 42, entry 1) and with a higher conversion, achieving a 41% conversion after 12 h. When the acylation was catalysed by CalA (entry 2) (*S*)-**2e** was obtained with 80% *ee* and a 42% conversion after eight hours (*E* = 16). The *p*-cyano derivative (±)-**3c** was a good substrate for both catalysts (entries 3 and 4). When using PSL-C, a 44% of (*S*)-**3e** with 90% *ee* was obtained after 12 h, in a resolution with a good enantioselectivity value (*E* = 40), whereas the resolution catalysed by CalA led to a 47% conversion after eight hours and a good selectivity (*E* = 30). The presence of an electron-donating group in the aromatic ring of the diol seemed to have a negative effect on both the activity (41% of (*S*)-**4e** after 24 h) and the selectivity (*E* = 32) of PSL-C (entry 3). This substrate was tested with CalA, but a low selectivity was observed (*E* = 11), in a kinetic resolution with a 36% conversion after 16 h, as shown in entry 6. A heteroaromatic 1,2-diol as (±)-**5d** was successfully resolved by PSL-C in MTBE. After 24 h, a 45% of (*S*)-**5e** with 88% *ee* was achieved in a process with good selectivity, as shown in entry 7. The use of CalA led to a 38% conversion after 16 h and moderate selectivity (*E* = 17, entry 8).

Regarding the aliphatic diol (±)-**6d**, in which the stereogenic center presents an aliphatic substituent, the enzymatic acylation in TBME at 30 °C using PSL-C afforded a very low enantioselectivity value (*E* = 7) in a very fast resolution, achieving a 45% of (*S*)-**6e** after 2 h. In view of this result, the isozyme A from *Candida antarctica* was tested, leading to a slower (*c* = 17% after 6 h), but slightly more selective process (*E* = 12) than with PSL-C (see entry 10). In order to improve the reaction selectivity, the PSL-C-catalysed resolutions were carried out using ethyl acetate as a less reactive acyl donor. After 6 h, a 15% of (*S*)-**6e** was obtained in a process with a low enantioselectivity (*E* = 10). Finally, the kinetic resolution in presence of vinyl acetate was conducted at 10 °C. After 4 h, a 17% of diol **6d** was converted into the acetate (*S*)-**6e** with 86% *ee*, but the *E* value was only increased to 16 (entry 12), indicating that this substrate was not appropriate for the biocatalysed acylation.

## 3. Materials and Methods

Unless otherwise noted, analytical grade solvents and commercially available reagents were used without further purification. Formaldehyde *tert*-butyl hydrazone [27] and organocatalyst **I** [28] were synthesized according to the literature. Racemic azocompounds (±)-**1**–**6b** [24,26] and 1,2-diols (±)-**1d** and (±)-**4,5d [29]** exhibited the same physical and spectral properties as described in the bibliography and the nuclear magnetic resonance (NMR) data of the 1,2-diols are shown in the Supplementary Information. *Pseudomonas cepacia* lipase PSL-C (1638 U/g) and lipases from *Pseudomonas fluorescens* (≥160 U/mg), *Rhizopus oryzae* (≥30 U/mg), *Aspergillus oryzae* (ca. 50 U/mg), *Burkholderia* sp. (≥160 U/mg), porcine pancreatic (≥20,000 U/mg), and *Mucor miehei* (≥4000 U/mg) were purchased from Sigma-Aldrich (Saint Louis, MO, USA). *Candida antarctica* lipase type B (CalB, Novozyme 435, 7300 propyl laureate units per gram) was obtained from Novozymes (Bagsvaerd, Denmark). *Candida antarctica* lipase A (CalA) was obtained from Codexis (Redwood City, CA, USA).

NMR spectra were recorded in CDCl_3_ [^1^H-NMR (300 MHz); ^13^C-NMR (75.4 MHz)] with the solvent peak used as the internal reference (7.26 and 77.0 ppm for ^1^H and ^13^C, respectively). High-resolution mass spectrometry (HRMS) analyses were performed with an Orbitrap ELITE instrument (Waltham, ThermoFisher, MA, USA). Column chromatography was performed on silica gel (Merck Kieselgel 60, Kenilworth, NJ, USA). Analytical thin layer chromatography (TLC) was performed on aluminum backed plates (1.5 × 5.0 cm) precoated (0.25 mm) with silica gel (Merck, Silica Gel 60 F254, Kenilworth, NJ, USA). The compounds were visualized by exposure to UV light or by dipping the plates into solutions of KMnO_4_ or vainilline stains followed by heating. HPLC analyses were performed on a Waters 2695 Instrument (Milford, MA, USA), equipped with a Waters 996 Photodiode Array Detector (Milford, MA, USA). To determine the enantiomeric excesses of diols (*R*)-**1**–**6d** and acetates (*S*)-**1**–**6e**, the following columns from Daicel (Tokyo, Japan) were employed: Chiralcel OD (25 × 0.46 cm) and Chiralpak AD-H (25 × 0.46 cm). The optical purity of the diols was measured after their derivatization to the corresponding acetates using acetic anhydride and pyridine in CH_2_Cl_2_. HPLC conditions and retention times are summarized in Table S1. The absolute configuration of the 1,2-diols (*R*)-**1**–**6d** and the acetates (*S*)-**1**–**6e** were established by comparison with the described values of the specific rotation for (*R*)-ethyl 2-benzyl-2,3-dihydroxypropanoate [23].

### 3.1. General Procedure for the Synthesis of Racemic Azocompounds (±)-***1***–***6b***

The corresponding α-ketoester **1**–**6a** (5.0 mmol) was dissolved at room temperature in toluene (8.0 mL) and *N*-*tert*-butyl formaldehyde hydrazone (10 mmol) was added. For the ethyl benzoylformate, catalyst **I** (0.5 mmol, 10 mol%) was added prior to the hydrazone. Reactions were stirred at room temperature for 24 h until consumption of the starting material (TLC). The solvent was eliminated under reduced pressure and the obtained crudes were purified by column chromatography using toluene/EtOAc mixtures as the eluent in order to obtain the corresponding racemic azocompounds (±)-**1**–**6b** with yields between 80 and 92%.

### 3.2. General Preparation of the Racemic 1,2-diols (±)-***1***–***6d*** Starting from Azocompounds (±)-***1***–***6b***

**Figure 2:**
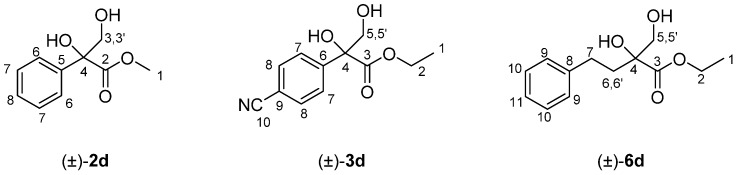
Structure and NMR assignation of the synthesized racemic 1,2-diols (±)-**2,3d** and **6d**.

The corresponding azocompound (±)-**1**–**6b** (4.0 mmol) was dissolved in Et_2_O (35 mL), cooled to 0 °C, and HCl 6.0 M (15 mL) was added. The reaction mixture was allowed to warm to room temperature and was stirred for 4 h, and then extracted with Et_2_O (2 × 15 mL) and CH_2_Cl_2_ (2 × 15 mL). The organic layers were dried over Na_2_SO_4_ and the solvent was eliminated in vacuo to create the crude α-hydroxyaldehydes (±)-**1**–**6c**, which were further reduced without purification. Tetrabutylamonium borohydride (560 mg, 2.0 mmol) was added to a solution of the obtained aldehyde in CH_2_Cl_2_ (15 mL) and the mixture was stirred at room temperature for 2 h. After evaporation of the organic solvent, the crude was purified by column chromatography using CH_2_Cl_2_/MeOH 97:3 as the eluent, yielding the racemic 1,2-diols (±)-**1**-**6d** (Figure 2).

(±)-Ethyl 2,3-dihydroxy-2-phenylpropanoate, (±)-**1d**: Yellow pale oil (395 mg, yield 47%). Spectroscopic data consistent with the literature [29].

(±)-Methyl 2,3-dihydroxy-2-phenylpropanoate, (±)-**2d**: Yellow pale oil (384 mg, yield 49%). ^1^H-NMR (300 MHz, CDCl_3_): δ (ppm) 7.53 (d, 2H, *J* = 8.0 Hz, H_7_), 7.33–7.24 (m, 3H, H_6_ and H_8_), 4.18 (d, 1H, *J* = 10.8 Hz, H_3_), 4.13 (bs, 1H, OH), 3.77 (s, 3H, H_1_), 3.68 (d, 1H, *J* = 10.8 Hz, H_3′_), 2.80 (bs, 1H, OH). ^13^C-NMR (75.4 MHz, CDCl_3_): δ (ppm) 174.2 (C_2_), 138.0 (C_5_), 128.5 (C_7_), 128.2 (C_6_), 125.3 (C_8_), 79.7 (C_4_), 68.3 (C_3_), 53.5 (C_1_). HRMS: *m*/*z* calcd. for C_10_H_12_NaO_4_ (M + Na^+^): 219.02626; found: 219.02628.

(±)-Ethyl 2-(4-cyanophenyl)-2,3-dihydroxypropanoate, (±)-**3d**: Colorless oil (517 mg, yield 55%). ^1^H-NMR (300 MHz, CDCl_3_): δ (ppm) 7.69 (d, 2H, *J* = 8.5 Hz, H_8_), 7.59 (d, 2H, *J* = 8.5 Hz, H_7_), 4.29–4.21 (m, 2H, H_2_), 4.15 (d, 1H, *J* = 11.4 Hz, H_5_), 4.07 (bs, 1H, OH), 3.66 (d, 1H, *J* = 11.4 Hz, H_5′_), 3.09 (bs, 1H, OH), 1.25 (t, 3H, *J* = 7.1 Hz, H_1_). ^13^C-NMR (75.4 MHz, CDCl_3_): δ (ppm) 172.6 (C_3_), 143.2 (C_6_), 132.2 (C_8_), 126.6 (C_7_), 118.5 (C_10_), 112.3 (C_9_), 79.4 (C_4_), 68.2 (C_5_), 63.3 (C_2_), 14.0 (C_1_). HRMS: *m*/*z* calcd. for C_12_H_13_NNaO_4_ (M + Na^+^): 258.0739; found: 258.0737.

(±)-Ethyl 2,3-dihydroxy-2-(4-methoxyphenyl)propanoate, (±)-**4d**: Colorless oil (432 mg, yield 45%). Spectroscopic data consistent with the literature [29].

(±)-Ethyl 2,3-dihydroxy-2-(tiophen-2-yl)propanoate, (±)-**5d**: White solid. m.p.: 76–78 °C (354 mg, yield 41%). Spectroscopic data consistent with the literature [29].

(±)-Ethyl 2-hydroxy-2-hydroxymethyl-4-phenylbutanoate, (±)-**6d**: Colorless oil (384 mg, yield 57%) ^1^H-NMR (300 MHz, CDCl_3_): δ (ppm) 7.20–7.12 (m, 2H, H_10_), 7.10–7.07 (m, 3H, H_9_ and H_11_), 4.15 (q, 2H, *J* = 7.0 Hz, H_2_), 3.98 (s, OH), 3.73 (d, 1H, *J* = 11.2 Hz, H_5_), 3.56 (d, 1H, *J* = 11.2 Hz, H_5′_), 2.91 (s, OH), 2.78–2.68 (m, 1H, H_6_), 2.43–2.33 (m, 1H, H_6′_), 1.98–1.78 (m, 2H, H_7_), 1.22 (t, 3H, *J* = 7.0 Hz, H_1_). ^13^C-NMR (75.4 MHz, CDCl_3_): δ (ppm) 175.0 (C_3_), 141.2 (C_8_), 128.5 (C_10_), 128.4 (C_9_), 126.0 (C_11_), 78.3 (C_4_), 68.0 (C_5_), 62.4 (C_2_), 36.6 (C_6_), 29.5 (C_7_), 14.2 (C_1_). HRMS: *m*/*z* calcd. for C_13_H_18_NaO_4_ (M + Na^+^): 261.1102; found: 261.1097.

### 3.3. General Synthesis of the Racemic Acetates (±)-***1***–***6e***

**Figure 3:**
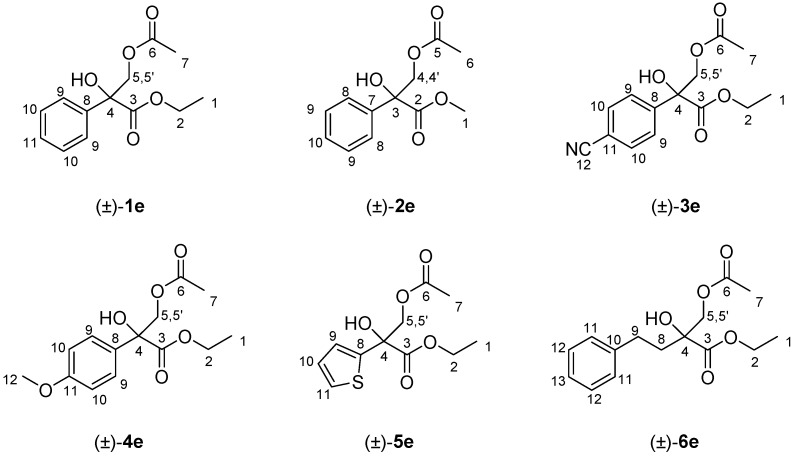
Structure and NMR assignation of the synthesized racemic acetates (±)-**1**–**6e**.

To a solution of the corresponding racemic 1,2-diol (±)-**1**-**6d** (0.2 mmol) in CH_2_Cl_2_ (2.0 mL), pyridine (18 μL, 0.22 mmol) and acetic anhydride (20 μL, 0.22 mmol) were added at room temperature. The reaction was stirred until disappearance of the starting material (TLC using hexane/EtOAc 7:3 as the eluent). The crude reaction was washed with HCl 1.0 N (2 × 2.0 mL), dried with Na_2_SO_4_, and the solvent was removed under reduced pressure to yield the corresponding racemic acetates (±)-**1**–**6e**, which were obtained after purification by column chromatography using *n*-hexane/EtOAc 7:3 as eluent (Figure 3).

(±)-Ethyl 3-acetoxy-2-hydroxy-2-phenylpropanoate, (±)-**1e**: Colorless oil (45.8 mg, yield 91%). ^1^H-NMR (300 MHz, CDCl_3_): δ (ppm) 7.57–7.54 (m, 2H, H_10_), 7.31–7.27 (m, 3H, H_9_ and H_11_), 4.68–4.64 (d, 1H, *J* = 11.3 Hz, H_5_), 4.30–4.15 (m, 3H, H_2_ and H_5′_), 3.84 (bs, 1H, OH), 2.00 (s, 3H, H_7_), 1.22 (t, 3H, *J* = 7.1 Hz, H_1_); ^13^C-NMR (75.4 MHz, CDCl_3_): δ (ppm) 172.8 (C_6_) 170.6 (C_3_), 137.6 (C_8_), 129.3 (C_10_), 128.5 (C_9_), 125.6 (C_11_), 79.2 (C_4_), 68.8 (C_2_), 62.9 (C_5_), 20.7 (C_7_), 14.1 (C_1_). HRMS: *m*/*z* calcd. for C_13_H_16_NaO_5_ (M + Na^+^): 275.0892; found: 275.0890.

(±)-Methyl 3-acetoxy-2-hydroxy-2-phenylpropanoate, (±)-**2e**: Colorless oil (44.2 mg, yield 93%). ^1^H-NMR (300 MHz, CDCl_3_): δ (ppm) δ (ppm) 7.55 (d, 2H, *J* = 8.2 Hz, H_9_), 7.31–7.27 (m, 3H, H_8_ and H_10_), 4.64 (d, 1H, *J* = 11.2 Hz, H_4_), 4.31 (d, 1H, *J* = 11.2 Hz, H_4′_), 3.77 (s, 3H, H_1_), 2.54 (bs, 1H, OH), 2.01 (s, 3H, H_6_). ^13^C-NMR (75.4 MHz, CDCl_3_): δ (ppm) 173.2 (C_5_), 170.6 (C_2_), 137.5 (C_7_), 128.7 (C_9_), 128.5 (C_8_), 125.6 (C_10_), 77.6 (C_3_), 68.8 (C_4_), 53.5 (C_1_), 20.8 (C_6_). HRMS: *m*/*z* calcd. for C_12_H_14_NaO_5_ (M + Na^+^): 261.0373; found: 261.0377.

(±)-Ethyl 3-acetoxy-2-(4-cyanophenyl)-2-hydroxypropanoate, (±)-**3e**: Colorless oil (48.7 mg, yield 88%). ^1^H-NMR (300 MHz, CDCl_3_): δ (ppm) 7.74–7.71 (d, 2H, *J* = 8.6 Hz, H_10_), 7.62–7.59 (d, 2H, *J* = 8.6 Hz, H_9_), 4.61 (d, 1H, *J* = 11.3 Hz, H_5_), 4.31–4.17 (m, 3H, H_2_ and H_5′_), 4.01 (bs, 1H, OH), 2.00 (s, 3H, H_7_), 1.23 (t, 3H, *J* = 7.1 Hz, H_1_). ^13^C-NMR (75.4 MHz, CDCl_3_): δ (ppm) 171.7 (C_6_), 170.4 (C_3_), 141.0 (C_8_), 132.2 (C_10_), 126.8 (C_9_), 119.0 (C_12_), 112.5 (C_11_), 77.3 (C_4_), 68.5 (C_5_), 63.5 (C_2_), 20.7 (C_7_), 14.0 (C_1_).HRMS: *m*/*z* calcd. for C_14_H_15_NNaO_5_ (M + Na^+^): 300.0842; found: 300.0849.

(±)-Ethyl 3-acetoxy-2-hydroxy-2-(4-methoxyphenyl)propanoate, (±)-**4e**: Colorless oil (50.8 mg, yield 90%). ^1^H-NMR (300 MHz, CDCl_3_): δ (ppm) 7.47 (d, 2H, *J* = 8.1 Hz, H_9_), 6.91 (d, 2H, *J* = 8.1 Hz, H_10_), 4.58 (d, 1H, *J* = 11.0 Hz, H_5_), 4.30–4.15 (m, 3H, H_2_ and H_5′_), 3.97 (bs, 1H, OH), 2.03 (s, 3H, H_7_), 1.30 (t, 3H, *J* = 7.0 Hz, H_1_). ^13^C-NMR (75.4 MHz, CDCl_3_): δ (ppm) 171.8 (C_6_), 169.7 (C_3_), 158.0 (C_11_), 131.1 (C_8_), 127.2 (C_9_), 111.0 (C_10_), 79.3 (C_4_), 68.0 (C_5_), 63.0 (C_2_), 57.2 (C_12_), 21.2 (C_7_), 14.0 (C_1_). HRMS: *m*/*z* calcd. for C_14_H_18_O_6_ (M^+^) 282.1103; found: 282.1098.

(±)-Ethyl 3-acetoxy-2-hydroxy-2-(tiophen-2-yl)propanoate, (±)-**5e**: Yellow pale oil (41.4 mg, yield 81%). ^1^H-NMR (300 MHz, CDCl_3_): δ (ppm) 7.25 (dd, 1H, *J* = 5.1, 1.3 Hz, H_11_), 7.10 (dd, 1H, *J* = 3.7, 1.3 Hz, H_10_), 6.98 (dd, 1H, *J* = 5.1, 3.7 Hz, H_9_), 4.56 (d, 1H, *J* = 11.1 Hz, H_5_), 4.33–4.19 (m, 3H, H_2_ and H_5′_), 2.91 (bs, 1H, OH), 2.00 (s, 3H, H_7_), 1.25 (t, 3H, *J* = 7.0 Hz, H1). ^13^C-NMR (75.4 MHz, CDCl_3_): δ (ppm) 171.8 (C_6_), 170.3 (C_3_), 141.7 (C_8_), 127.2 (C_11_), 125.9 (C_10_), 125.0 (C_9_), 76.4 (C_4_), 69.2 (C_5_), 63.2 (C_2_), 20.7 (C_7_), 13.9 (C_1_). HRMS: calcd. for C_11_H_14_NaO_4_S (M + Na^+^): 281.0456; found: 281.0454.

(±)-Ethyl 2-(acetoxymethyl)-2-hydroxy-4-phenylbutanoate, (±)-**6e**: Colorless oil (47.6 mg, yield 85%). ^1^H-NMR (300 MHz, CDCl_3_): δ (ppm) 7.21–7.18 (m, 2H, H_12_), 7.13–7.07 (m, 3H, H_11_ and H_13_), 4.19–4.08 (m 4H, H_2_, H_5_ and H_5′_), 3.51 (bs, 1H, OH), 2.79–2.70 (m, 1H, H_8_), 2.45–2.35 (m, 1H, H_8′_), 1.99–1.90 (m, 5H, H_7_ and H_9_), 1.21 (t, 3H, *J* = 7.1 Hz, H_1_). ^13^C-NMR (75.4 MHz, CDCl_3_): δ (ppm) 173.9 (C_6_), 170.5 (C_3_), 141.0 (C_10_), 128.4 (C_11_), 126.1 (C_12_), 125.7 (C_13_)_,_ 76.0 (C_4_), 68.9 (C_5_), 62.4 (C_2_), 36.8 (C_8_), 29.3 (C_9_), 20.7 (C_7_), 14.2 (C_1_). HRMS: *m*/*z* calcd. for C_15_H_20_NaO_5_ (M^+^): 303.1204; found: 303.1203.

### 3.4. General Procedure for the Biocatalyzed Acylation of the Racemic 1,2-diols (±)-***1***–***6d***

Unless otherwise stated, vinyl acetate (0.9 mmol) was added to a solution of the racemic diol (±)-**1**–**6d** (0.3 mmol) in TBME (2.0 mL) containing the PSL-C (30 mg) and Na_2_CO_3_ (0.25 mmol). Reactions were stirred at 30 °C at 220 rpm and monitored by TLC using *n*-hexane/EtOAc 7:3 as the eluent. Once finished, the lipase was filtered, washed with TBME (2 × 2 mL), and the solvent was evaporated under reduced pressure. The crude mixture was purified by column chromatography using *n*-hexane/EtOAc 7:3 as the eluent in order to obtain the (*R*)-1,2-diols **1**–**6d** and the (*S*)-acetates **1**–**6e**, which were analysed by HPLC at the conditions described in Table S1 for the determination of the optical purities. (*R*)-**1d**: 37.8 mg, 60% yield; [α]${}_{D}^{21}$ = −2.9 (*c* = 0.6, CHCl_3_, 47% *ee*); (*S*)-**1e**: 23.0 mg, 32% yield; [α]${}_{D}^{21}$ = +10.8 (*c* = 1.0, CHCl_3_, 92% *ee*). (*R*)-**2d**: 33.8 mg, 57% yield; [α]${}_{D}^{21}$ = −3.8 (*c* = 0.5, CHCl_3_, 55% *ee*); (*S*)-**2e**: 24.1 mg, 35% yield; [α]${}_{D}^{21}$ = +12.1 (*c* = 0.8, CHCl_3_, 92% *ee*). (*R*)-**3d**: 50.5 mg, 71% yield; [α]${}_{D}^{21}$ = −4.2 (*c* = 1.0, CHCl_3_, 32% *ee*); (*S*)-**3e**: 19.4 mg, 22% yield; [α]${}_{D}^{21}$ = +9.8 (*c* = 1.2, CHCl_3_, 93% *ee*). *(R)-***4d**: 51.6 mg, 72% yield; [α]${}_{D}^{21}$ = −1.7 (*c* = 0.4, CHCl_3_, 30% *ee*); (*S*)-**4e**: 16.9 mg, 20% yield; [α]${}_{D}^{21}$ = +6.8 (*c* = 1.2, CHCl_3_, 92% *ee*). (*R*)-**5d**: 38.1 mg, 59% yield; [α]${}_{D}^{21}$ = −2.7 (*c* = 0.75, CHCl_3_, 50% *ee*); (*S*)-**5e**: 23.2 mg, 30% yield; [α]${}_{D}^{21}$ = +10.2 (*c* = 1.05, CHCl_3_, 90% *ee*). (*R*)-**6d**: 34.0 mg, 48% yield; [α]${}_{D}^{21}$ = −3.8 (*c* = 0.5, CHCl_3_, 50% *ee*); and (*S*)-**6e**: 33.6 mg, 40% yield, [α]${}_{D}^{21}$ = +5.6 (*c* = 1.2, CHCl_3_, 62% *ee*).

## 4. Conclusions

A set of aromatic and non-aromatic 1,2-diols containing an ester moiety were prepared in a three-step procedure with moderate yields from the corresponding α-ketoesters. These functionalized racemic 1,2-diols were tested in lipase-catalysed acetylations. After optimization of the reaction conditions, we achieved good activities and selectivities in the resolution of aromatic 1,2-diols by employing the *Pseudomonas cepacia* lipase (PSL-C) in *tert*-butyl methyl ether as the solvent. This biocatalyst showed a higher selectivity for the preparation of chiral (*R*)-1,2-diols and (*S*)-acetates containing unsubstituted aromatic rings or presenting electron-withdrawing groups, whereas the reactions were slower and slightly less selective for aromatic substrates with electron-donating groups or heteroaromatic systems. PSL-C could be recycled for five reactions without appreciable loss in its biocatalytic properties, thus resulting in a promising biocatalyst for the preparation of optically active 1,2-diols.

## Figures and Tables

**Table 1:**
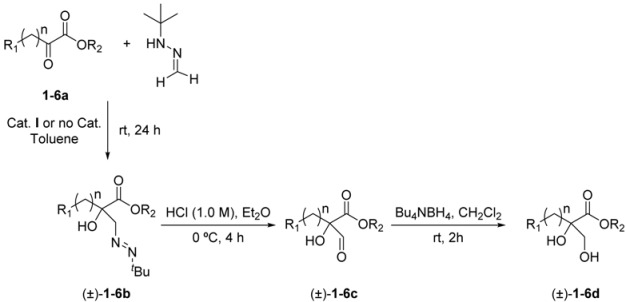
Synthesis of racemic 1,2-diols (±)-**1**–**6d** in a three-step procedure starting from the commercially available α-ketoesters **1**–**6a**.

Entry	R_1_	R_2_	n	Yield (±)-1–6b ^a^	Yield (±)-1–6d ^a^
1	Ph	Et	0	(±)-**1b**, ^b^ 87	(±)-**1d**, 47
2	Ph	Me	0	(±)-**2b**, 80	(±)-**2d**, 49
3	4-CN-Ph	Et	0	(±)-**3b**, 92	(±)-**3d**, 55
4	4-OMe-Ph	Et	0	(±)-**4b**, 85	(±)-**4d**, 45
5	2-Thiophenyl	Et	0	(±)-**5b**, 90	(±)-**5d**, 41
6	H	Et	2	(±)-**6b**, 85	(±)-**6d**, 57

^a^ For reaction conditions, see Materials and Methods. ^b^ Reaction performed with 10 mol% of catalyst **I**.

**Table 2:**
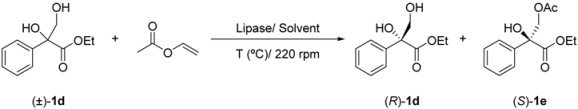
Lipase-catalysed acylation of *rac*-ethyl 2,3-dihydroxy-2-phenylpropanoate (**1d**) at different reaction conditions.

Entry	Lipase	Solvent	T (°C)	t (h)	*c* (%) ^a^	*ee* 1d (%) ^b^	*ee* 1e (%) ^c^	*E* ^d^
1	CalB	Toluene	30	4	23	11	37	2
2	CalA	Toluene	30	12	32	37	77	12
3	PSL-C	Toluene	30	20	32	40	86	20
4	PSF	Toluene	30	20	41	35	50	4
5	BSL	Toluene	30	20	38	42	69	8
6	*R. oryzae*	Toluene	30	20	23	22	75	9
7	*A. oryzae*	Toluene	30	20	16	11	57	4
8	PPL	Toluene	30	24	48	67	73	13
9	*M. miehei*	Toluene	20	24	13	12	78	9
10	PSL-C	TBME	30	12	42	62	91	41
11	CalA	TBME	30	6	41	54	79	17
12	PPL	TBME	30	12	38	50	77	13
13	PSL-C	1,4-Dioxane	30	20	23	25	86	17
14	PSL-C	THF	30	20	6	6	87	15
15	PSL-C	DIPE	30	12	45	67	83	22
16	PSL-C	TBME	15	24	43	67	91	43
17	PSL-C ^e^	TBME	30	24	36	52	91	37
18	PSL-C ^f^	TBME	30	48	34	47	91	34

^a^ Conversion, *c* = *ee*_s_/(*ee*_s_ + *ee*_p_). ^b^ Enantiomeric excesses were determined by high performance liquid chromatography (HPLC) after acetylation in presence of acetic anhydride in pyridine. ^c^ Determined by HPLC. ^d^ Enantioselectivity (*E*) value, *E* = ln[1 − *c*(1 + *ee*_p_)]/ln [1 − *c*(1 − *ee*_p_)]. ^e^ Reaction performed with isopropenyl acetate as acyl donor. ^f^ Reaction performed with ethyl acetate as acyl donor.

**Table 3:**
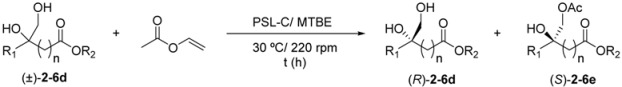
PSL-C catalysed kinetic resolution of racemic diols **2**–**6d** in *tert*-butyl methyl ether (TBME) at 30 °C using vinyl acetate as the acyl donor.

Entry	Substrate	Lipase	R_1_	R_2_	n	t (h)	*c* (%) ^a^	*ee* 2-6d (%) ^b^	*ee* 2-6e (%) ^c^	*E* ^d^
1	(±)-**2d**	PSL-C	Ph	Me	0	12	41	63	91	42
2	(±)-**2d**	CalA	Ph	Me	0	8	42	59	80	16
3	(±)-**3d**	PSL-C	4-CN-Ph	Et	0	12	44	71	90	40
4	(±)-**3d**	CalA	4-CN-Ph	Et	0	8	47	77	86	30
5	(±)-**4d**	PSL-C	4-OMe-Ph	Et	0	24	41	62	89	32
6	(±)-**4d**	CalA	4-OMe-Ph	Et	0	16	36	42	72	11
7	(±)-**5d**	PSL-C	2-Thiophenyl	Et	0	24	45	73	88	33
8	(±)-**5d**	CalA	2-Thiophenyl	Et	0	16	38	47	83	17
9	(±)-**6d**	PSL-C	Ph	Et	2	2	45	50	62	7
10	(±)-**6d**	CalA	Ph	Et	2	6	17	17	82	12
11 ^e^	(±)-**6d**	PSL-C	Ph	Et	2	6	15	14	80	10
12 ^f^	(±)-**6d**	PSL-C	Ph	Et	2	4	17	18	86	16

^a^ Conversion, *c* = *ee*_s_/(*ee*_s_ + *ee*_p_). ^b^ Enantiomeric excesses were determined by HPLC after acetylation in presence of acetic anhydride in pyridine. ^c^ Determined by HPLC. ^d^ Enantioselectivity (*E*) value, *E* = ln[1 − *c*(1 + *ee*_p_)]/ln [1 − *c*(1 − *ee*_p_)]. ^e^ Reaction carried out with ethyl acetate as acyl donor. ^f^ Reaction carried out at 10 °C.

## Supplementary Materials

The following are available online at http://www.mdpi.com/1420-3049/23/7/1585/s1, Table S1: HPLC analyses; and assignment of ^1^H and ^13^C-NMR of synthesized compounds.
