# Correction: de Gonzalo, G. Lipase Catalysed Kinetic Resolution of Racemic 1,2-Diols Containing a Chiral Quaternary Center. *Molecules* 2018, *23*, 1585

**DOI:** 10.3390/molecules23102503

**Published:** 2018-09-29

**Authors:** Gonzalo de Gonzalo

**Affiliations:** Departamento de Química Orgánica, Universidad de Sevilla, c/Profesor García González 1, 41012 Sevilla, Spain; gdegonzalo@us.es; Tel.: +34-954559997

The authors wish to make the following corrections to their paper [1]. The authors are sorry to report that the absolute configurations of the 1,2-diols (**1**–**5d**) as well as the acetates (**1**–**5e**) obtained in this paper are inverted. In order to establish the absolute configuration of these compounds, specific rotation values should be compared with those described in [2] for (*R*)-**1d**, (*R*)-**4d** and (*R*)-**5d**, instead of the value provided in [3] for (*R*)-ethyl 2-benzyl-2,3-dihydroxypropanoate. This compound is the benzylic analogue of 1,2-diols **1**–**5d**. Compound **6d** (ethyl 2-hydroxy-2-hydroxymethyl-4-phenylbutanoate) presents the chiral quaternary center bound to an aliphatic carbon instead of an aromatic one. Thus, specific rotation values cannot be compared with the ones given in [2], and only the relative configurations of **6d** and **6e** are indicated. Consequently, the authors wish to make the following corrections to the paper:

In the enzymatic kinetic resolutions of 1,2-diols containing the chiral center bound to an aromatic carbon atom, (*S*)-1,2-diols *(S*)-**1**–**5d** and (*R*)-acetates (*R*)-**1**–**5e** are obtained. With regard to substrate **6d**, the 1,2-diol achieved was (−)-**6d**, whereas the acetate obtained was (+)-**6e**. 

Thus, we replace all over the manuscript (*R*)-**1**–**5d** with (*S*)-**1**–**5d**, (*S*)-**1**–**5e** with (*R*)-**1**–**5e**, (*R*)-**6d** with (−)-**6d** and (*S*)-**6e** with (+)-**6e**. In page 4, replace “(±)-**1e**” with “(±)-**1d**”, “(*S)*-acetates **2**–**6e**” with “(*R)*-acetates **2**–**5e**”.

In page 6 we change “The absolute configuration of the 1,2-diols (*R*)-**1**–**6d** and the acetates (*S*)-**1**–**6e**” with “The absolute configuration of the 1,2-diols (*S*)-**1**–**5d** and the acetates (*R*)-**1**–**5e**”, and “(*R*)-ethyl 2-benzyl-2,3-dihydroxypropanoate [23].” to “(*R*)-ethyl 2,3-dihydroxy-2-phenylpropanoate [(*R*)-**1d**], (*R*)-ethyl 2,3-dihydroxy-2-(4-methoxyphenyl)propanoate [(*R*)-**4d**], and (*R*)-ethyl 2,3-dihydroxy-2-(tiophen-2-yl)propanoate, [(*R*)-**5d**] in reference [26].”

The scheme of Table 2 is to be replaced:

**Table 2.** Lipase-catalysed acylation of *rac*-ethyl 2,3-dihydroxy-2-phenylprpanoate (**1d**) at different reaction conditions. 
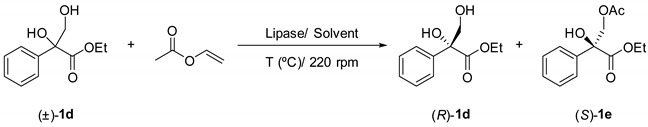
 with

**Table 2.** Lipase-catalysed acylation of *rac*-ethyl 2,3-dihydroxy-2-phenylpropanoate (**1d**) at different reaction conditions. 
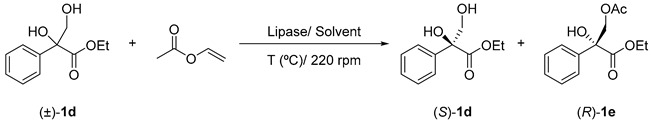
 with

The scheme of Table 3 is also to be replaced:

**Table 3.** PSL-C catalysed kinetic resolution of racemic diols **2**–**6d** in *tert*-butyl methyl ether (TBME) at 30 °C using vinyl acetate as the acyl donor. 
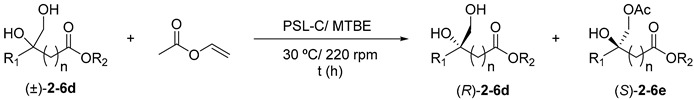
 with

**Table 3.** PSL-C catalysed kinetic resolution of racemic diols **2**–**6d** in *tert*-butyl methyl ether (TBME) at 30 °C using vinyl acetate as the acyl donor.

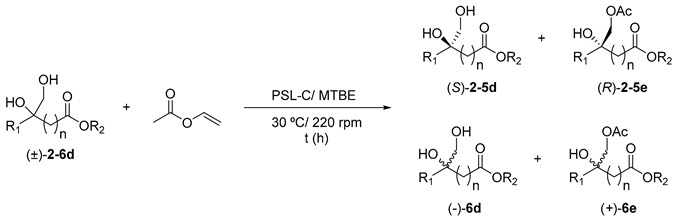



The author would like to apologize for any inconvenience caused to the readers. The manuscript will be updated and the original will remain online on the article webpage, with a reference to this Correction.
