# Supplementary material for: Lipase Catalysed Kinetic Resolution of Racemic 1,2-Diols Containing a Chiral Quaternary Center
Source: Molecules. 2018 Jun 29;23(7):1585. doi: 10.3390/molecules23071585 (PMC6100382; doi:10.3390/molecules23071585)
Supplement: Supplementary file 1 [file molecules-23-01585-s001.pdf]

Lipase catalyzed kinetic resolution of racemic 1,2-diols containing a chiral quaternary center

Gonzalo de Gonzalo

Supporting Information

Contents:

1. HPLC analyses……………………………………………………………..………………..S2
2. NMR signals of the known compounds………………………………………………….S2

Molecules 2018, 23, 1585; doi:10.3390/molecules23071585 www.mdpi.com/journal/molecules

1. HPLC Analyses

For the determination of the enantiomeric excesses of compounds (*R*)-**1-6c** and (*S*)-**1-6d** (Table S1), the following columns were employed: column A: Chiralcel OD (0.46 cm x 25 cm) and column B: Chiralpak AD-H (0.46 cm x 25 cm), both from Daicel. The optical purity of the chiral 1,2-diols **1-6c** is determined after acetylation to the acetates **1-6d**.

Table S1. Determination of enantiomeric excesses of acetates **1-6d** by HPLC.

Compounds	Column	Flow rate (mL min^{-1})	T (ºC)	Eluent[a]	Retention time [min]
**1d**	A	1.0	30	*n*-hexane-IPA 99:1	15.3 (*R*); 19.0 (*S*)
**2d**	A	1.0	30	*n*-hexane-IPA 99:1	13.9 (*R*); 17.2 (*S*)
**3d**	B	1.0	30	*n*-hexane-IPA 95:5	19.5 (*S*); 30.1 (*R*)
**4d**	B	1.0	30	*n*-hexane-IPA 95:5	14.9 (*S*); 20.0 (*R*)
**5d**	B	1.0	30	*n*-hexane-IPA 95:5	11.2 (*S*); 16.8 (*R*)
**6d**	A	1.0	30	*n*-hexane-IPA 99:1	19.0 (*R*); 21.1 (*S*)

[a] All the experiments were performed with isocratic eluent.

2. NMR Signals of the known compounds

(±)-Ethyl 2,3-dihydroxy-2-phenylpropanoate, (±)-**1d**: ^{1}H-RMN (300 MHz, $CDCl_3$): δ (ppm) 7.60 (d, 2H, *J* = 8.2 Hz), 7.39-7.30 (m, 3H), 4.37-4.25 (m, 3H), 4.13 (bs, 1H, OH), 3.75 (d, 1H, *J* = 11.2 Hz), 2.56 (bs, 1H, OH), 1.31 (t, 3H, *J* = 7.1 Hz). ^{13}C-RMN (75.4 MHz, $CDCl_3$): δ (ppm) 173.6 (C=O), 138.1 (C_{Ar}), 128.3 ($2CH_{Ar}$), 128.2 ($2CH_{Ar}$), 125.3 (CH_{Ar}), 79.4 (C), 68.2 (CH_2), 62.8 (CH_2), 13.9 (CH_3).

(±)-Ethyl 2,3-dihydroxy-2-(4-methoxyphenyl)propanoate, (±)-**4d**: ^{1}H-RMN (300 MHz, $CDCl_3$): δ (ppm) 7.50 (d, 2H, *J* = 8.5 Hz), 6.89 (d, 2H, *J* = 8.5 Hz), 4.34-4.25 (m, 2H), 4.21 (d, 1H, *J* = 11.2 Hz), 4.10 (bs, 1H, OH), 3.80 (s, 3H), 3.72 (d, 1H, *J* = 11.2 Hz), 2.57 (bs, 1H, OH), 1.30 (t, 3H, *J* = 7.0 Hz). ^{13}C-RMN (75.4 MHz, $CDCl_3$): δ (ppm) 173.8 (C=O), 159.4 (C_{Ar}), 130.2 (C_{Ar}), 126.4 ($2CH_{Ar}$), 113.7 ($2CH_{Ar}$), 79.1 (C), 68.2 (CH_2), 62.7 (CH_2), 55.2 (CH_3), 14.0 (CH_3).

(±)-Ethyl 2,3-dihydroxy-2-(tiophen-1-yl)propanoate, (±)-**5c**: ^{1}H-RMN (300 MHz, $CDCl_3$): δ (ppm) 7.25 (dd, 1H, *J* = 5.4, 1.7 Hz), 7.10 (dd, 1H, *J* = 3.4, 1.7 Hz), 6.98 (dd, 1H, *J* = 5.4, 3.4 Hz), 4.37-4.26 (m, 3H), 4.13 (d, 1H, *J* = 11.5 Hz), 3.80 (d, 1H, *J* = 11.5 Hz), 2.34 (bs, 1H, OH), 1.33 (t, 3H, *J* = 7.0 Hz). ^{13}C-RMN (125 MHz, CDCl3): δ (ppm) 172.7 (C=O), 142.2 (C_{Ar}), 127.1 (CH_{Ar}), 125.4 (CH_{Ar}), 124.4 (CH_{Ar}), 78.3 (C), 69.0 (CH_2), 63.2 (CH_2), 13.9 (CH_3).

Molecules 2018, 23, 1585; doi:10.3390/molecules23071585 www.mdpi.com/journal/molecules
